# Imaging of primary human hepatocytes performed with micron-sized iron oxide particles and clinical magnetic resonance tomography

**DOI:** 10.1111/j.1582-4934.2008.00343.x

**Published:** 2008-04-10

**Authors:** Nathanael Raschzok, Mehmet H Morgul, Jens Pinkernelle, Florian W R Vondran, Nils Billecke, Nora N Kammer, Gesine Pless, Michaela K Adonopoulou, Christian Leist, Lars Stelter, Ulf Teichgraber, Ruth Schwartlander, Igor M Sauer

**Affiliations:** a General, Visceral and Transplantation Surgery, Charité-Campus Virchow, Universitätsmedizin BerlinBerlin, Germany; b Istanbul Faculty of Medicine, Istanbul UniversityIstanbul, Turkey; c Radiology, Charité-Campus Virchow, Universitätsmedizin BerlinBerlin, Germany

**Keywords:** MRI, MPIO, hepatocyte transplantation, cell detection, cell labelling

## Abstract

Transplantation of primary human hepatocytes is a promising approach in certain liver diseases. For the visualization of the hepa-tocytes during and following cell application and the ability of a timely response to potential complications, a non-invasive modality for imaging the transplanted cells has to be established. The aim of this study was to label primary human hepatocytes with micron-sized iron oxide particles (MPIOs), enabling the detection of cells by clinical magnetic resonance imaging (MRI). Primary human hepatocytes isolated from 13 different donors were used for the labelling experiments. Following the dose-finding studies, hepatocytes were incubated with 30 particles/cell for 4 hrs in an adhesion culture. Particle incorporation was investigated *via* light, fluorescence and electron microscopy, and labelled cells were fixed and analysed in an agarose suspension by a 3.0 Tesla MR scanner. The hepatocytes were enzymatically resuspended and analysed during a 5-day reculture period for viability, total protein, enzyme leakage (aspartate aminotransferase [AST], lactate dehydrogenase [LDH]) and metabolic activity (urea, albumin). A mean uptake of 18 particles/cell could be observed, and the primary human hepatocytes were clearly detectable by MR instrumentation. The particle load was not affected by resuspension and showed no alternations during the culture period. Compared to control groups, labelling and resuspension had no adverse effects on the viability, enzyme leakage and metabolic activity of the human hepatocytes. The feasibility of preparing MPIO-labelled primary human hepatocytes detectable by clinical MR equipment was shown *in vitro*. MPIO-labelled cells could serve for basic research and quality control in the clinical setting of human hepatocyte transplantation.

## Introduction

Transplantation of primary human hepatocytes is a promising approach for treating certain liver diseases like metabolic disorders as well as chronic or acute liver failure [1, 2]. So far, liver transplantation still remains the gold standard for most liver diseases. However, its applicability is restricted by the shortage of donor organs. Cell transplantation is less invasive than whole-organ transplantation, provides an option for treating multiple patients with single-donor organs and allows for the utilization of autologous tissue for hepatocyte isolation [3, 4].

Hepatocyte transplantation is based on the application of cells in suspension [5]. The preparation of primary human hepatocytes for cell transplantation requires either isolation from freshly resected liver tissue, thawing of cryopreserved cells or resuspension of temporary cultivated cells. Applying the cells *via* the portal vein, the splenic artery or into the splenic parenchyma can lead to reorganization in the spleen (hepatization) or integration into the liver parenchyma [1]. However, a method for monitoring the processes during and following hepatocyte transplantation is still lacking. In clinical trials of hepatocyte transplantation, either the biopsies were taken from the target organ or the donor hepatocytes were visualized by radioisotope imaging [6, 7]. Both methods show limitations regarding the safety for the patient and the long-term analysis of the transplanted cells and cannot fully address the concern of distinct localization of the cells. Recent studies have shown that magnetic resonance imaging (MRI) might be a suitable option to solve these problems [8]. MRI enables the non-invasive assessment and visualization of anatomy with very high spatial resolution and excellent soft tissue contrast. Compared to other non-invasive visualization strategies, such as computer tomography or scintigraphy, MRI requires no gamma- or X-ray exposition. Therefore, this method provides advanced safety to the patient and enables real-time and repetitive examinations as well as intra-operative cell tracking.

Most strategies for MRI of single cells are based on labelling with superparamagnetic iron oxide particles (SPIOs) [8–11]. These particles are commercially available in different sizes and some are already approved for clinical use [12]. Experiments on cell labelling using nano-sized SPIOs have shown their detectability after *in vivo* accumulation by clinical MR equipment [13, 14]. Recently, the first experiences with *in vitro* MRI of primary human hepatocytes using SPIOs have been reported [15]. Although SPIOs are widely used for cell imaging, it has to be considered that large numbers of nano-sized particles must be incorporated into the targeted cells to enable their detection by MRI [16, 17]. Further limitations are the slow incorporation of unmodified SPIOs and their instability, which can cause a loss in detection and cytotoxicity [16].

In order to achieve fast labelling, high safety of the particle load and detectability of the labelled cells on a single-cell level, micron-sized iron oxide particles (MPIO) were introduced to cellular imaging [18]. Various cell types have been labelled with these particles, including macrophages and different human tumour cell lines, clearly proving the detectability of MPIO-labelled cells on a single-cell level [16–24]. However, the feasibility of using MPIO-labelled primary human hepatocytes for cell transplantation has not yet been evaluated.

This work describes a protocol for the preparation of MPIO-labelled primary human hepatocytes suitable for cell transplantation. Particle incorporation and detectability of the cells in an agarose suspension by clinical MR instrumentation were evaluated, and the effects of the particle load as well as the preparation procedure for transplantation were investigated *in vitro*.

## Materials and methods

### Primary human hepatocytes

#### Isolation and culture conditions

Following ethical and institutional guidelines and after informed consent of the tissue donors, samples were collected from a total of 13 patients undergoing partial hepatectomy (mean age of donor 52.0 ± 5.8 years). The specimens (20–40 g) were taken from the resected liver tissue and transferred to the laboratory under sterile conditions. Primary human hepatocytes were isolated using a modified two-step collagenase perfusion technique, as already described by Katenz *et al.*[25]. Following the isolation procedure, cell counts and viability were determined *via* the trypan blue exclusion test.

Freshly isolated hepatocytes were seeded on collagen-coated 6- and 96-well culture plates (Sarstedt, Nürnbrecht, Germany) and 8-well culture slides (BioCoat, Bedford, MA, USA) at concentrations of 1 × 10^6^, 0.05 × 10^6^ and 0.2 × 10^6^ viable cells. Cells were cultivated in Williams' medium E (Biochrom AG, Berlin, Germany), supplemented with 1 μM insulin, 1 μm dexamethason/fortecortin, 100 U/ml penicillin, 100 μg/ml streptomycin, 1 mM sodium pyruvate, 15 mM N-2-hydroxyethylpiper-azine-N′-2-ethane sulfonic acid buffer (HEPES), 4 mM L-glutamine and 10% foetal calf serum (FCS). After the attachment phase, the cells were washed with phosphate-buffered saline (PBS; PAA, Pasching Germany) and supplied with fresh medium. The medium was changed every 24 hrs, and the supernatant was collected and stored (−80 C) for further analysis.

For resuspension, primary human hepatocytes in the 6-well plates were used. The cells were washed and detached from the culture plates using 0.25/0.02% trypsin/ethylenediaminetetraacetic acid (EDTA) solution (Biochrom AG). The cell suspension was collected, and cell counts and viability were determined. Total recovery of the resuspend-ed cells was calculated: number of living cells after resuspension × 100/number of initially seeded living cells. For reculture, again 1 × 10^6^ and 0.05 × 10^6^ living cells per well were seeded into 6- and 96-well plates, respectively.

#### MPIO labelling

The superparamagentic MPIOs (ME03F/8064; Bangs Laboratories, IN USA) are divinyl benzene polymer encapsulated microspheres with a stated size of 1.63 μm. The particles contain a magnetite iron oxide component (42.5%) and are Dragon Green fluorescent-labelled (480/520 nm) within a polymer encapsulation. For cell labelling, the culture medium was removed and replaced with a particle solution at the respective concentration. After labelling, the cells were washed three times with PBS to remove free and loosely bound particles.

### Design of the study

#### Particle uptake

In order to find the optimal conditions for MPIO labelling and visualization of primary human hepatocytes by MR equipment, the concentration of the particles and the time of incubation were investigated with cells from 4 of the 13 donors.

Following a 24-hrs pre-culture period, the hepatocytes were incubated with increasing concentrations of MPIOs (10, 20, 30 or 40 particles/cell) for 18 hrs at 37 C. The initial time of incubation of 18 hrs was based on the experiences of Shapiro *et al.* with the murine hepatocytes [16]. The number of incorporated particles was determined by light microscopy and the cells were scanned in an agarose suspension using 3.0 Tesla MR instrumentation. Immunofluorescence and electron microscopic observations were performed to confirm the intracellular localization of the particles. The minimum number of incorporated particles showing a strong signal at low signal-to-noise ratio was determined, and the minimum incubation concentration for this amount of particles was chosen for further experiments.

After determining the required particle load, experiments were performed to reduce the incubation time. Human hepatocytes can be successfully labelled when allowed to attach for only 18 hrs. Thus, the time of pre-culture was reduced (data not shown). The hepatocytes were incubated for 2, 4, 6 and 8 hrs at a concentration of 30 particles/cell. The minimum incubation time resulting in adequate particle uptake was chosen for further experiments. Each experiment was repeated three times.

#### Impact on cell integrity and metabolic activity

The hepatocytes from 9 of the 13 donors were used for studying the impact of MPIO labelling and resuspension on cell integrity and metabolic activity. Isolated cells from each donor were divided into four groups and cultivated for 6 days.

- Group A: control without MPIOs, medium change 24 hrs after isolation.
- Group B: incubation with 30 particles/cell for 4 hrs at 37 C (after 18-hr pre-culture), medium change 24 hrs after isolation.
- Group C: control without MPIOs, resuspension and reseeding 24 hrs after isolation.
- Group D: incubation with 30 particles/cell for 4 hrs at 37 C (after 18-hr pre-culture), resuspension and reseeding 24 hrs after isolation.

The hepatocytes were characterized during a 5-day period (culture days 2–6) for mitochondrial activity, total protein, enzyme leakage (aspartate aminotransferase [AST], lactate dehydrogenase [LDH]) and metabolic activity (urea, albumin). Furthermore, the particle load was determined.

### Analysis

#### Light microscopy

Light microscopy (Axiovert 40CFL; Zeiss, Oberkochen, Germany) was performed on culture days 1, 2, 4 and 6. Pictures were acquired using a digital camera (QICAM FAST 1394; QIMAGING, Surrey, British Columbia, Canada). For the determination of the particle load, the pictures at a 200× magnification were divided into a raster of 20 fields using a graphic software (Graphic Converter X, Universal Binary V5.9; Lemkesoft GmbH, Peine, Germany). Five fields per picture were randomly chosen (Microsoft Excel for Mac 2004, V11.3.6; Microsoft Corporation, Redmond, WA, USA), and the particles were counted twice by two independent investigators. The labelling efficiency was calculated as the number of labelled cells × 100/total number of cells.

#### Fluorescence microscopy

Following labelling and washing, the cells were fixed using 4% paraformaldehyde for 10 min. at room temperature and permeabilized using ice-cold methanol for 20 min. at −20°C on culture day 1. The cells were stained for cytoplasmic proteins and the nucleus by incubation with a primary antibody against human cytokeratine 18 (Santa Cruz Biotechnology Santa Cruz, CA, USA), secondary antibody Indocarbocyanine (JacksonImmunoResearch, Newmarket, Suffolk, United Kingdom) and 4′,6′-diamidino-2-phenylindole (Sigma-Aldrich, Steinheim, Germany) for 1 hr, respectively. The analyses were performed using a filter set and a fluorescence lamp.

#### Electron microscopy

On the first day of the culture period, the labelled hepatocytes were resuspended and left to sediment. The pellets were fixed in 2.5% glutaraldehyde and the cells were post-fixed using 2% osmium tetroxide. The hepatocytes were embedded in 2% agar, dehydrated through ascending alcohol and infiltrated overnight in an Epon-containing solution. The samples were placed in plastic capsules and polymerized (60°C, 48 hrs). Ultra-thin sections were transferred onto a 200-mesh copper grids and stained with 4% aqueous uranyl acetate and Reynolds lead citrate. The sections were imaged using an electron microscope (EM 906; Zeiss).

#### Mitochondrial activity and biochemical parameters

The mitochondrial activity of the resuspended cells was determined on culture days 2 and 6 in 96-well plates using the CellTiter 96Aqueous One solution Cell Proliferation Assay (Promega, Mannheim, Germany). The biochemical parameters were analysed from the culture supernatants in 6-well plates using commercially available test kits from culture day 2 to day 6 on a daily basis. Albumin was analysed only on days 2, 3, 4 and 6. AST and LDH enzyme activities were measured (NobiFlow GOT-IFCC and NobiFlow LDH-L [IFCC]). Urea formation was detected *via* an enzyme-based detection kit (NobiFlow Harnstoff-UV; all HITADO, Möhnesee, Germany), and albumin synthesis was assessed using an enzyme-linked immunosorbent assay kit (Albuwell II; WAK-Chemie, Steinbach Germany). Protein concentration was determined with a bicinchroninic acid (BCA) assay from whole-cell lysates (Interchim, Mont Lucon, France) at the end of the culture period.

#### Magnetic resonance imaging

The cell samples for MRI were prepared using labelled and unlabelled cells from the same donors. Immediately after labelling, on the first day of the culture, the cells were resuspended in the culture medium and embedded in 1% agarose (Eurogentec, Seraing, Belgium). The cryotubes (1.8 ml) were half-filled with 1 ml of agarose as a support layer. For each group, 50 μl of cell suspension containing 1000 cells was mixed with 200 μl of agarose and was placed on the support layer. After the cell layer became solid, the tubes were sealed with a top layer of agarose. As controls, the samples containing native cells (without particles) or MPIOs (without cells) were diluted in 250 μl of agarose and prepared in the same manner.

The cell samples were investigated by high-resolution imaging on a clinical MR scanner at a field strength of 3.0 Tesla (Signa 3T94; General Electrics Healthcare, Milwaukee, WI, USA) using a circularly polarized surface coil with a diameter of 2 cm (Rapid Biomedical, Wuerzburg, Germany). A T2*-weighted, two-dimensional gradient-echo pulse sequence was used for image acquisition. Selection of a field of view of 20 mm, a matrix size of 256 × 256 pixels and a slice thickness of 0.8 mm lead to a nominal voxel resolution of 78 μm × 78 μm × 800 μm. The imaging sequence was employed at a repetition time of 200 msec, an echo time of 25 msec. and a flip angle of 20. The number of excitations acquired was kept at 12, resulting in a total imaging time of 5.10 min. The signal-to-noise ratio (SNR) was measured in four different regions of the image background with varying distance to the coil. SNR was calculated as (signal -noise)/mean error of noise using Image J 1.38 for Windows (Wayne Rasbad, National Institute of Health, Bethesda, MD, USA).

#### Statistical analysis

For each parameter, the measurements were repeated at least three times. A statistical analysis (two-tailed Student's t-test and one-way anova) was carried out using SPSS software version 13 for Windows (SPSS, Inc., Chicago, IL, USA). The graphical presentations of the data were generated using Deltagraph (Version 5.0.1 for Apple Macintosh OS X, SPSS, Inc./Red Rock Software, Salt Lake City, UT, USA). A *P* value less than or equal to 0.05 was considered as statistically significant. The values are expressed as mean ± standard error of mean (S.E.M.).

## Results

### Concentration and time dependency of particle uptake

**Fig. 1:**
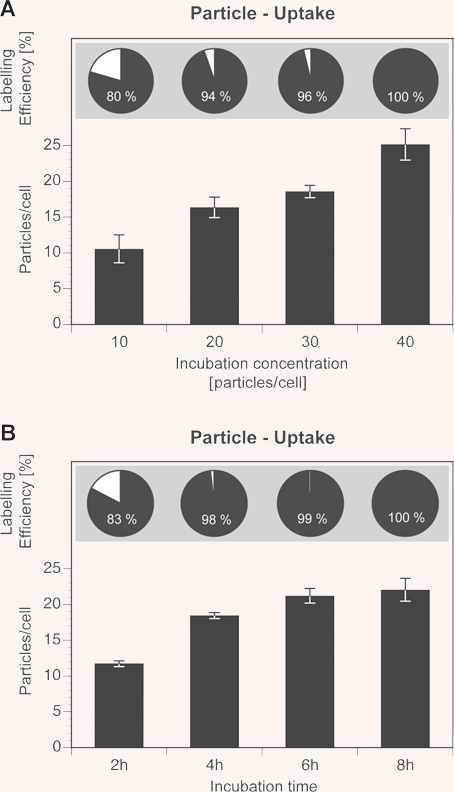
Concentration and time dependency of particle uptake. (**A**) 18-hr incubation of primary human hepatocytes (24-hr pre-culture period) with increasing concentrations of MPIOs (10, 20, 30 and 40 particles/cell) resulted in an increase in particle uptake and labelling efficiency (80.00 ± 7.33%, 94.00 ± 2.79%, 96.00 ± 1.06%, 100%, respectively). (**B**) Time dependency of the particle uptake was investigated at a concentration of 30 MPIOs/cell (18-hr pre-culture period). Labelling efficiency: 83.00 ± 5.48%, 98.00 ± 1.85%, 99.00 ± 0.76%, 100%. Data are given as mean ± S.E.M.

**Fig. 2:**
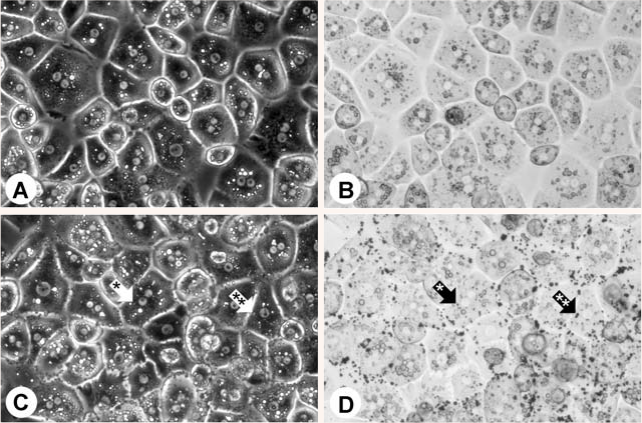
Light microscopy of native (**A**, **B**) and labelled hepatocytes (**C**, **D**). MPIOs were clearly visible within the cytoplasm of the hepatocytes, both as single particles (*) and as clusters (**). Morphological differences were not detected between native and labelled cells. Pictures were taken at 200x magnification using phase contrast (**A**, **C**) and transmitted light (**B**, **D**).

The dose-determining experiments clearly revealed the dependency of the particle uptake on the concentration of MPIOs during incubation. When the hepatocytes were incubated with increasing concentrations of MPIOs for 18 hrs, the number of particles per cell increased from 10 to 25 (Fig. 1A). The labelling efficiency showed similar characteristics (79.50 ± 7.33%–100.00%). As assessed by light microscopy, the particles were distributed throughout the entire cytoplasm of the cells and partially formed clusters of about three to five particles (Fig. 2). The incubation with MPIOs for 2–8 hrs at a concentration of 30 particles/cell showed a dependency of both particle uptake and labelling efficiency on the incubation time. After 18 hrs of pre-culture, the particle load increased from 12 to 22, while the labelling efficiency reached a plateau of approximately 100% after 4 hrs of incubation (Fig. 1B). The pre-culture period affected the particle uptake and the labelling efficiency. When the hepatocytes were incubated with 30 particles/cell after being allowed to attach for 24 hrs, a mean uptake of 18 particles/cell and a labelling efficiency of 96.25 ± 1.06% was achieved. The same particle uptake and a labelling efficiency of 98.15 ± 1.85% was reached by cells pre-cultured for 18 hrs and incubated for 4 hrs with the same amount of particles.

**Fig. 3:**
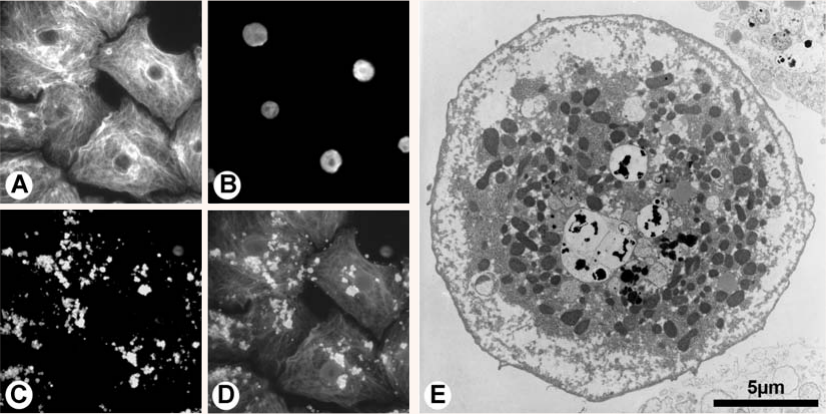
Fluorescence and electron microscopy of MPIO-labelled primary human hepatocytes. Cells were stained against cytokeratine 18 (**A**) and nuclei (**B**). Dragon Green labelled MPIOs (**C**) were distributed throughout the cytoplasm. In the overlay (**D**), cytokeratine 18 is illustrated as red, nuclei as blue and MPIOs as green. Electron microscopy of single hepatocytes proved the intracellular localization of the particles, both as single particles and as clusters (**E**).

Fluorescence microscopy revealed the intracellular localization of the particles and showed no morphological alternations due to the particle uptake (Fig. 3A–D). Electron microscopy confirmed the incorporation of the particles, both as single particles and as clusters. The encasement of the particles within the cytoplasmic vesicles as well as their iron core was clearly detectable (Fig. 3E).

**Fig. 4:**
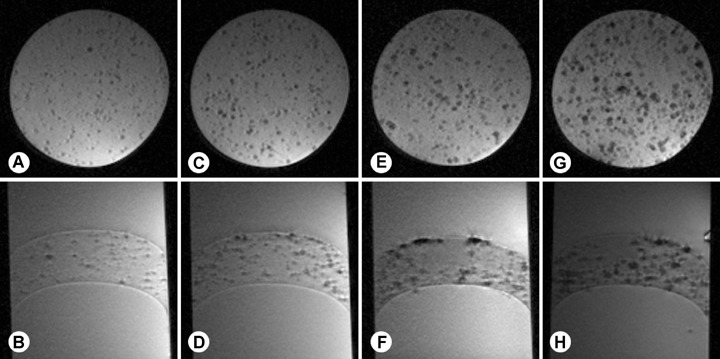
Agarose samples of hepatocytes with increasing particle load were investigated using T2*-weighted, two-dimensional gradient echo pulse sequence by 3.0 Tesla in sagittal and axial slices. Cells containing 10 ± 2 particles/cell (**A**, **B**) or 16 ± 1 particles/cell (**C**, **D**) showed areas of slight hypo-density. Preparations of cells containing 18 ± 1 particles (**E**, **F**) or 25 ± 2 particles (**G**, **H**) displayed punctuate signal extinctions that clearly contrast against the high-signal image background caused by the aqueous agarose medium. Imaging parameters were as follows: repetition time/echo time/flip angle = 200 msec/25 msec./20°. Spatial resolution was 78 μm × 78 μm × 800 μm requiring scan times of 5.10 min.

**Fig. 5:**
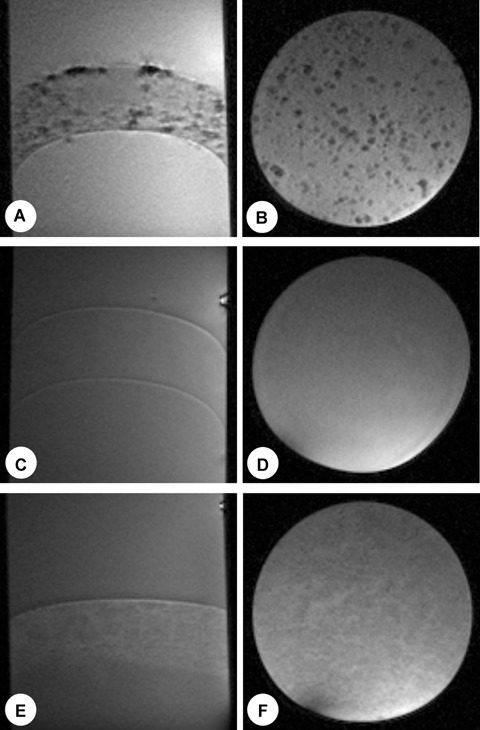
Cells labelled with 18 ± 1 MPIOs/cell were clearly detectable both in sagittal and axial slices (**A**, **B**) at a concentration of 1000 cells/250 μl. The same number of non-labelled cells (**C**, **D**) or a correlating number of MPIOs suspended in agarose (**E**, **F**) showed no detectable distinct signal changes. Imaging parameters were the same as in Fig. 4.

In order to investigate the number of particles per cell required to enable detection by MR instrumentation, the samples of hepatocytes with increasing particle content embedded in agarose were analysed. The signal extinctions of the labelled cells correlated to the number of incorporated particles and increased with the particle load. The images of cells containing 18 ± 1 (Fig. 4E and F) or 25 ± 2 particles (Fig. 4G and H) displayed distinct signal extinctions at low SNR (28.82 ± 5.27 and 30.66 ± 5.37) that clearly contrast against the high-resolution image background caused by the aqueous agarose medium. On the basis of the previously published MR images of MPIO-labelled cells in the agarose suspension [16–24], an uptake of about 17–20 particles per cell (Fig. 5A and B) was assumed to be sufficient for cell detection with MR equipment at 3.0 Tesla. This particle load was achieved by incubating the hepatocytes with 30 particles/cell. The agarose samples containing cells with 10 ± 2 (Fig. 4A and B) or 16 ± 1 particles per cell (Fig. 4C and D) showed lower signal extinctions at higher SNR (45.09 ± 8.41 and 38.38 ± 7.12) and were, therefore, estimated as not suitable for cell detection in the agarose samples. The MR images of the agarose samples containing an identical number of unla-belled cells (Fig. 5C and D) or an equivalent amount of MPIOs without cells (Fig. 5E and F) had a uniform appearance and revealed no distinct signal extinctions. According to these data, the concentration of 30 particles/cell and an incubation time of 4 hrs resulting in a mean particle load of 18 were defined as the standard conditions for further cell-labelling experiments.

### Particle uptake and retention

**Fig. 6:**
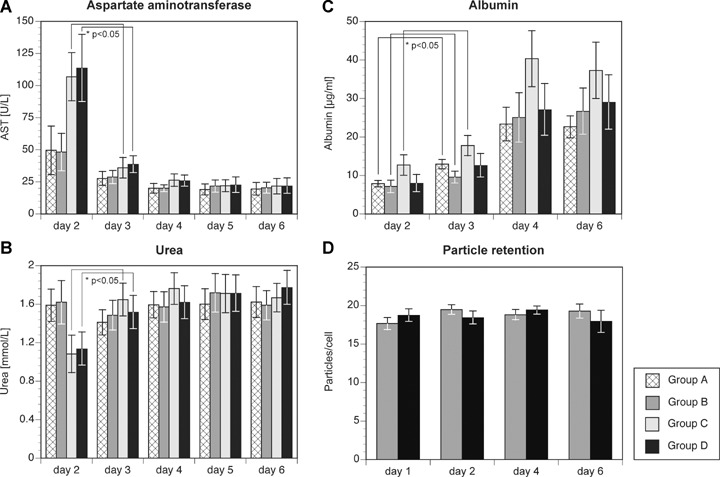
Biochemical parameters (**A**-**C**) and particle retention (**D**) of labelled primary human hepatocytes. Group A: unlabelled/adherent, group B: labelled/adherent, group C: unlabelled/resuspended and group D: labelled/resuspended. Data are given as mean ± S.E.M. Significant differences when compared to the previous culture day: *P < 0.05. There were no statistically significant differences in the particle load between group B (adherent) and group D (resuspended) during the entire culture period (*P* > 0.05).

After incubation of cells with MPIOs for 4 hrs, 97.34 ± 0.70% of the cells were labelled with an average of 18 ± 1 particles per cell (Fig. 6D). Twenty-four hours after labelling, the mean particle load of the adherent (group B) and resuspended hepatocytes (group D) was 19 ± 1 and 18 ± 1, respectively. At the end of the culture period, the cells of group B contained an average of 19 ± 1 particles and group D contained 18 ± 1 particles. There were no statistically significant differences in the particle load between the adherent group and the resuspension group during the entire culture period. Cell viability of the freshly isolated primary human hepatocytes was 75.22 ± 2.24%. By trypsin treatment, 52.17 ± 3.79% of unlabelled (group C) and 55.11 ± 6.75% of labelled (group D) initially seeded viable hepatocytes could be resuspended, achieving a viability of 68.93 ± 3.24% and 72.53 ± 3.54%, respectively. There was no evidence that the particles had a negative effect on the success of the trypsin treatment. The freshly isolated cells showed the typical morphological appearance of primary human hepatocytes (Fig. 2A and B). They presented a polygonal shape, granular cytoplasm with vesicular inclusions and one or more nuclei. The labelling procedure caused no alternations in the morphology of the hepatocytes (Fig. 2C and D). Shortly after incubation, the particles were situated on the cell membrane; at later time-points, they were detected mostly in the peri-nuclear cytoplasm, both as single particles as well as clusters. Over the entire culture period, no morphological differences were seen among the four groups.

### Cell integrity and metabolism

The mitochondrial activity of resuspended cells was not affected by the particle incorporation. Statistically significant differences were not detected in the resuspension groups between the unlabelled and labelled cells at culture day 2 (group C: 2.51 ± 0.10, group D: 2.32 ± 0.33) and culture day 6 (group C: 2.28 ± 0.27, group D: 2.60 ± 0.30). No statistical differences were detected among the groups concerning the total protein at the end of the culture period, verifying the same amount of cells in the different groups (data not shown). In both resuspension groups, a peak in AST release was observed 24 hrs after trypsin treatment, followed by a rapid decrease from 106.8 U/L (group C) and 113.4 U/L (group D) to 36.0 U/L and 38.4 U/L on the next day, respectively (Fig. 6A). In the meantime, the AST levels of the adherent groups decreased from 49.5 U/L (group A) and 48.2 U/L (group B) to 27.7 U/L and 28.7 U/L, respectively. Throughout the rest of the culture period, the AST release in all groups was constant. LDH concentrations of both resuspension groups increased between day 2 and day 3 to the levels of the control groups (C: 11.2 U/L to 14.6 U/L; D: 9.7 U/L to 14.8 U/L), while the other groups showed no alternations. From day 3 to the end of the culture period, there were no significant differences between the groups concerning the LDH release. A significant increase in the LDH release could only be observed in the resuspension groups from day 5 to day 6. Concerning the particle load, no differences in the cell damage parameters were detected between the labelled and unlabelled cells from day 2 to day 6. Urea synthesis in the resuspended hepa-tocytes was significantly lower 24 hrs after resuspension, but showed a rapid increase thereafter (Fig. 6B). From day 3 until day 6, the mean urea synthesis in the groups varied between 1.4 mM/l and 1.8 mM/l per day and showed no significant differences among the groups. Albumin secretion increased during the culture period in all groups and did not show significant differences within the four groups at any time-point of measurement (Fig. 6C), but the albumin production of group C tended to be higher than group D at each sample point. Differences between the labelled and unla-belled cells concerning their metabolic activity were not detectable between day 2 and day 6.

## Discussion

To visualize the processes between the application and engraftment of the human hepatocytes, a non-invasive modality for imaging the transplanted cells has to be established. Invasive methods like his-tological detection of single cells are not suitable for continuous monitoring of dynamic processes such as cell application and engraftment. The use of radioisotope imaging is associated with exposition to radiation and shows limitations concerning long-term monitoring and resolution. A very promising approach was recently published by Landis et al. They developed genetically modified hepatocytes expressing the creatine kinase gene, permitting ^31^P MR spectroscopic imaging. However, *ex vivo* gene transfer methods are not approved for clinical applications yet [26]. MRI is the most promising technique for non-invasive visualization of single cells under clinical conditions, offering unique spatial resolution and allowing repetitive examinations of the patient without radiation expositions. High-resolution imaging of single cells over a prolonged period of time by clinical MR equipment could serve the analysis of basic mechanisms in cell-based regenerative therapy concepts and may be a valuable tool for quality control in the clinical setting of hepatocyte transplantation: monitoring transplanted cells is the basis for timely response to potential complications during cell application and engraftment, for example, unwanted systemic distribution [27].

The visualization of human hepatocytes via MRI requires their labelling with SPIOs [8]. In previous studies, nano-sized MagForce particles were used for this purpose [15]. Since the results were not sufficient for the detection of hepatocytes on a single-cell level, the efforts were focussed on cell labelling with MPIOs. Compared to nano-sized particles, MPIOs contain a larger iron core that has a great impact on the local magnetic field. The cells can be labelled without modification of the particles or the use of transfection agents, since the low density of functional carboxyl groups on the polymer shell of the MPIOs enables a fast cellular uptake [17]. The potential of MPIOs for high-resolution imaging of single cells has been proven in various cell types [16–24], and Shapiro *et al.* showed the feasibility of detecting single MPIO-labelled murine hepatocytes *in vivo* using an MR scanner at 7.0 Tesla [20].

In this study, the adherent primary human hepatocytes were successfully labelled with MPIOs within a 4-hr incubation period. The particle load was stable throughout the entire culture period of 6 days, and the intracellular localization of the particles was proven by fluorescence and electron microscopy, excluding possible misinterpretations caused by extracellularly attached particles. The hepatocytes containing 17–20 particles per cell were clearly detectable by clinical MR equipment at 3.0 Tesla and showed strong signal extinctions *in vitro* on the background of the agarose gel. The MR images of MPIO-labelled hepatocytes are in concordance with the previously published data of other types of MPIO-labelled cells in agarose suspension [16–24]. The signal extinctions generated by MPIO-labelled hepatocytes were stronger than the images of SPIO-labelled cells when acquired using the same MR equipment and voxel resolution. Correlation between signal extinctions and labelled cells in agarose suspension at this voxel resolution has already been proven [9]. To increase the signal-to-noise ratio, a circularly polarized surface coil was used in this study. Clinical phased-array coils consist of 32 or more of such elements, gaining SNR by parallel imaging. However, high SNR levels are realized close to the array, and the sensitivity drops significantly towards the deeper regions of interest. Further studies are required to translate the results of *in vitro* imaging of MPIO-labelled hepatocytes to clinical practice of cell transplantation.

Our monitoring concept for human hepatocyte transplantation is based on labelling a fraction of hepatocytes prior to transplantation. Therefore, the cells have to be temporarily cultivated to ensure a fast *in vitro* labelling procedure, due to the onset of the cells' metabolic activity, and require resuspension, thereafter. As generally applied for enzymatic resuspension, a combination of trypsin and EDTA was used in this study to detach the adherent cells following the labelling procedure. By this treatment, more than half of the initially seeded hepatocytes could be successfully detached, without a negative effect on their viability and particle load. The loss of cells might be attributed to a partially inadequate adhesion of the hepatocytes after initial seeding and to the resistance of some cells to enzymatic resuspension. This has been seen in previous studies as well: Grossmann *et al.* trypsinized primary human hepatocytes for hepatocyte transplantation after a 3-day culture period [28]. They could only gain a total number of about 33% of the initially seeded cells. By preparing MPIO-labelled hepatocytes for cell transplantation, the loss of a distinct portion of cells could be tolerated, since an MRI follow-up should not require the labelling of all transplanted cells. Solving the logistical problem of preparing a sufficient number of labelled cells, imaging of a small fraction of cells can be sufficient for drawing conclusions about the localization of the overall amount of transplanted cells.

In order to evaluate the potential harm of the labelling and resuspension procedure, we analysed the standard parameters for hepatocyte damage and metabolism [25]. Cultured primary hepa-tocytes undergo dedifferentiation as a function of the cultivation time [29]. Previous experiments showed that the morphology and metabolic activity of the primary human hepatocytes rapidly decrease after a period of 5–7 days [25]. Therefore, the observation time was limited to 6 days. The enzymes AST and LDH were used as indicators for the membrane integrity of the hepatocytes. Both enzymes are located in the cytoplasm of the hepatocytes – while AST is additionally placed in the hepatocyte's mitochondria – and are released in cases of a severe loss of membrane integrity [30, 31]. We observed high AST levels in the resuspended cells at the beginning of the culture period, followed by a rapid decrease from day 2 to day 3. Afterwards, there were no detectable differences between the resuspension and control groups. The LDH release from the resuspension group reached the levels in control groups within 1 culture day. From days 3 to 6, this parameter showed no abnormalities when compared to the controls, but the resuspension groups displayed an increase in their LDH leakage between days 5 and 6. Since the culture period was stopped at day 6, a further increase cannot be excluded by this study. However, the other investigated parameters did not correlate with the increase in LDH leakage at culture day 6. Initially, the high levels of AST were associated with damage of a distinct fraction of the cells by resuspension and decreased to a baseline level within 1 day. Since the isolation process harmed the hepato-cytes of the control groups as well, their AST levels were also elevated at culture day 2. The constantly low level of AST during the rest of the culture period indicated the integrity of the resuspend-ed cells, and the analysis of the mitochondrial activity confirmed these results. Similarly, alternations in the metabolic activity of the labelled and unlabelled hepatocytes were not observed. The cells reached the level of urea synthesis of the control groups at culture day 2; afterwards, the urea synthesis was similar in both groups. The albumin production of the hepatocytes was comparable in all groups and increased during the entire culture period. The slightly elevated albumin levels of the unlabelled and resuspended cells hint towards a higher metabolic activity of these cells compared to the labelled and resuspended cells. The analysis of total protein at the end of the culture period indicated a stable number of meta-bolically active cells within all culture groups. Adverse effects of the particle load on the human hepatocytes were not observed in this study. This observation is consistent with previous studies on MPIO labelling, in which degradation of intracellularly incorporated MPIOs has not been observed [17–19, 22]. As supposed by other authors, the stability and biocompatibility of the polymer shell of the MPIOs is considered to prevent degradation by intra-cellular lysosomes, reducing the risk of oxidative stress and cell damage by iron toxicity [16, 22, 24]. However, further steps are necessary prior to a clinical application of MPIO-labelled hepato-cytes. Long-term studies are currently performed in a large animal model to verify our first results and investigate the biocompatibil-ity and possible immunogenicity of labelled hepatocytes. Furthermore, these studies address the question whether MPIO-labelled primary hepatocytes are detectable by clinical MRI *in vivo*. Faster labelling techniques and improvement in the resuspension protocol or – more preferably – a concept for temporary suspension culture of primary human hepatocytes could reduce the loss of cells during preparation for transplantation. Currently, a protocol for labelling the human hepatocytes in the Rotary Cell Culture System (Synthecon, Houston, TX, USA), providing zero-gravity conditions combined with continuous oxygen supply, is under development. This approach may circumvent the time-consuming, and to some extent, harmful process of detaching and resuspending the labelled cells.

In conclusion, we present the first study on the preparation of MPIO-labelled primary human hepatocytes. The feasibility of the intracellular incorporation of the particles as well as the detection of the labelled primary human hepatocytes by clinical MR equipment could be shown *in vitro*. MPIO-labelled cells could serve as a valuable tool for both basic research in cell-based regenerative therapies and quality control in the clinical setting of human hepa-tocyte transplantation.
